# RGD peptide modified RBC membrane functionalized biomimetic nanoparticles for thrombolytic therapy

**DOI:** 10.1007/s10856-023-06719-1

**Published:** 2023-04-12

**Authors:** Zichen Xu, Jinxia Huang, Tao Zhang, Wenfeng Xu, Xiaoling Liao, Yi Wang, Guixue Wang

**Affiliations:** 1 Key Laboratory of Biorheological Science and Technology of Ministry of Education, State and Local Joint Engineering Laboratory for Vascular Implants, Bioengineering College of Chongqing University, Chongqing, 400030 China; 2 Chongqing Key Laboratory of Nano/Micro Composite Material and Device, School of Metallurgy and Materials Engineering, Chongqing University of Science and Technology, Chongqing, 401331 China; 3 College of Basic Medical Sciences, Chongqing Medical University, Chongqing, 400016 China

**Keywords:** Thrombus, Biomimetic, RBC membrane, RGD peptide, Targeted thrombolysis

## Abstract

In recent years, the fabrication of nano-drug delivery systems for targeted treatment of thrombus has become a research hotspot. In this study, we intend to construct a biomimetic nanomedicine for targeted thrombus treatment. The poly lactic-co-glycolic acid (PLGA) was selected as the nanocarrier material. Then, urokinase and perfluoro-n-pentane (PFP) were co-loaded into PLGA by the double emulsification solvent evaporation method to prepare phase change nanoparticles PPUNPs. Subsequently, the RGD peptide-modified red blood cell membrane (RBCM) was coated on the surface of PPUNPs to prepare a biomimetic nano-drug carrier (RGD-RBCM@PPUNPs). The as-prepared RGD-RBCM@PPUNPs possessed a “core-shell” structure, have good dispersibility, and inherited the membrane protein composition of RBCs. Under ultrasound stimulation, the loaded urokinase could be rapidly released. In vitro cell experiments showed that RGD-RBCM@PPUNPs had good hemocompatibility and cytocompatibility. Due to the coated RGD-RBC membrane, RGD-RBCM@PPUNPs could effectively inhibit the uptake of macrophages. In addition, RGD-RBCM@PPUNPs showed better thrombolytic function in vitro. Overall, the results suggested that this biomimetic nanomedicine provided a promising therapeutic strategy for the targeted therapy of thrombosis.

**Graphical Abstract:**

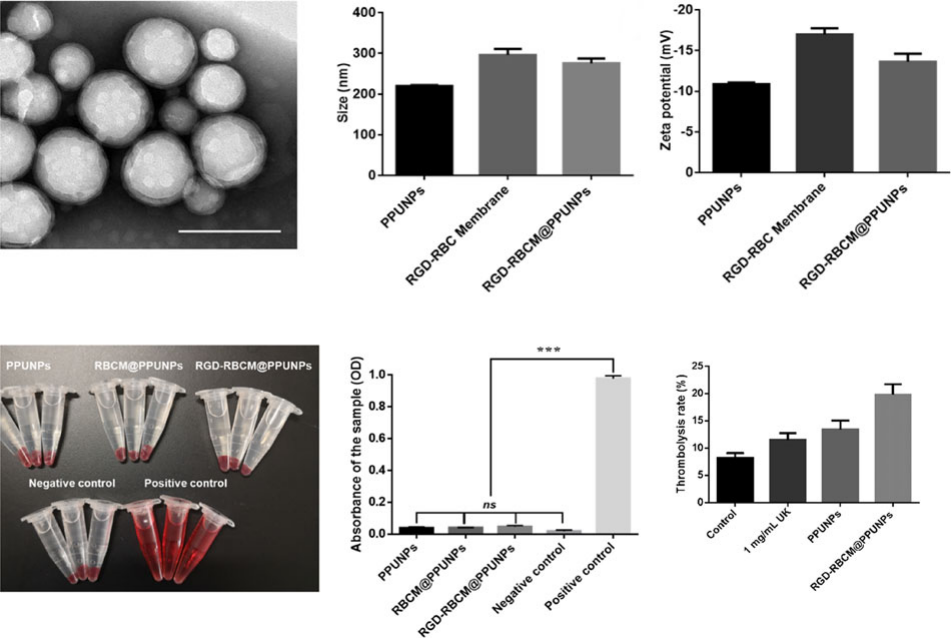

## Introduction

Thrombosis, or the formation of a malignant blood clot, causes many cardiovascular diseases, such as stroke, myocardial infarction and venous thromboembolism, and is one of the main causes of death [1–4]. Thrombosis is usually formed by abnormal accumulation of platelets and fibrin due to vascular injury or activation of coagulation factors [5, 6]. In clinical, surgery and antithrombotic drug therapy are commonly used. However, due to the high risk, high cost, and invasiveness of surgery, antithrombotic drug therapy is usually the first option of treatment [7, 8]. Conventional antithrombotic drugs mainly include thrombolytics, anticoagulants, and antiplatelet agents [9]. The main antiplatelet agents and anticoagulants used for thrombolytic therapy are heparin, urokinase (UK), tissue plasminogen activator (tPA), recombinant tPA (rtPA), and streptokinase (SK) [10, 11]. However, these agents are poorly targeted, affect normal hemostasis, and carry a risk of adverse bleeding complications. In addition, most antithrombotic drugs have short half-lives and require repeated administration, resulting in additional treatment costs and risks [8]. Therefore, in order to reduce the side effects of drugs, prolong the half-life of drugs, and obtain better therapeutic effects, it is necessary to develop drugs that can be delivered to the thrombus site.

Over the past few decades, nanomedicine has provided effective strategies for disease treatment. Nanomedicines can prolong the circulation time in the blood, realize the targeted delivery of therapeutic drugs [12, 13], improve the efficacy, and minimize the systemic toxicity of drugs [14–16]. In recent years, the application of nanotechnology in the treatment of thrombus has been extensively studied, and many NPs-based drug delivery systems have been investigated to treat thrombosis [17]. Generally, currently designed nanodrugs can reduce the size of thrombus by inducing hemolysis, fibrin degradation, and self-deformation of blood clots with the loaded drugs under certain conditions. While, these synthetic nano-drug delivery systems are usually recognized as foreign substances by the phagocytic system of the human body, which restricts targeted drug delivery.

Recently, an international frontier research team has developed a technology to directly coat cell membrane on the surface of NPs, which not only maintains the characteristics of the NPs, but also gives NPs with unique biological functions, such as inhibiting the clearance of monocyte phagocytic system and specific targeting of disease site [18, 19]. Until now, various different types of membrane have been used to fabricate cell membrane coated biomimetic NPs [20]. Among them, the red blood cell (RBC) membrane is an attractive choice to enwrap NPs because of its outstanding accessibility (the most abundant cell in blood), good biocompatibility and biodegradability [21, 22]. In addition, targeting ligands can be modified on the RBC membrane through insert and covalent bonding methods [23]. Therefore, using ligands modified RBC membranes to encapsulate NPs can improve the long-acting circulation and targeted drug delivery functions of NPs.

Ultrasound-responsive polymer materials have received attention in recent years [24]. The effects of ultrasound interacting with biological systems include thermal and non-thermal effects [25]. Cavitation is a non-thermal effect, causing the bubble to grow suddenly under the action of ultrasound [26]. Ultrasound can be precisely focused on the disease site to trigger drug release from NPs [27]. Stuart Ibsen and colleagues prepared a liposome system that simultaneously encapsulates drugs and perfluoro hexane (PFH). Under ultrasound trigger, the PFH can produce microbubbles within liposomes and release the loaded drug [28]. Wang et al. reported a nanodrug carrier composed of polymethacrylic acid (PMAA) nanocapsules which encapsulate PFH liquids. This nanodrug carrier possessed the function of ultrasound imaging and drug-controlled release [29]. Thereby, co-loading drugs and PFH in polymer NPs can realize the drugs’ controllable release under an ultrasound trigger.

In this study, we constructed an RGD peptide modified RBC membrane-coated biomimetic NPs for thrombolytic therapy. First, PLGA was selected as nanocarrier material, and then UK and PFP were co-loaded to prepare UK-loaded phase-change NPs (PPUNPs). Then, the RBC membrane was extracted by hypotonic hemolysis, and DSPE-PEG-RGD was modified on the RBC membrane by the insert method. Finally, the RGD-modified RBC membrane was coated on the surface of PPUNPs by co-extrusion technology to prepare the thrombolytic biomimetic NPs (RGD-RBCM@PPUNPs). The physicochemical properties, in vitro safety and function of RGD-RBCM@PPUNPs were investigated.

## Materials and methods

### Materials

PLGA (MW: 90000, LA: GA = 50:50) was purchased from Jinan Daigang Bioengineering Co., Ltd. (Jinan, China); dichloromethane was purchased from Chengdu Kelong Chemical Co., Ltd. (Chengdu, China); UK and polyvinyl alcohol (PVA-210, MW: ~67,000) were purchased from Shanghai Aladdin Biochemical Technology Co., Ltd. (Shanghai, China); PFP was purchased from Strem Chemicals, Inc (Massachusetts, USA); Isopropanol was purchased from Chongqing Sichuan East Chemical Co., Ltd. (Chongqing, China); Bradford protein concentration assay kit, DAPI staining solution and 4% paraformaldehyde fix solution was purchased from Shanghai Biyuntian Biotechnology Co., Ltd. (Shanghai, China); DSPE-PEG-FITC, DSPE-PEG-RGD (the MW of PEG is 2000) were purchased from Hunan Huateng Pharmaceutical Co., Ltd. (Hunan, China); CCK-8 kit was purchased from Dojindo Institute of Chemistry (Japan); The native tissue/cell lysate kit was purchased from Beijing Solarbio Science & Technology Co.,Ltd. (Beijing, China); Dulbecco’s modified Eagle’s medium (DMEM), 1640 medium, fetal bovine serum (FBS), and penicillin-streptomycin were purchased from Gibco (USA).

### Animals

Male C57BL/6 mice (18–20 g, 6–8 weeks) and Male New Zealand white rabbit (1.5–2 kg) were obtained from the Army Military Medical University in Chongqing, China. They were housed in standard mouse and rabbit cages with ad libitum access to water and food, respectively. All animal-related procedures complied with the China Council on Animal Care and Chongqing University protocol for animal use. All ethical guidelines for experimental animals were followed.

### Preparation of UK-loaded phase change NPs

PLGA nanoparticles loaded both urokinase and perfluoro-n-pentane (PPUNPs) were prepared by a double-emulsion solvent evaporation method (W1/O/W2). First, 50 mg of PLGA was completely dissolved in 2 mL of dichloromethane as the organic phase (O). 200 μL UK solution (2 mg/mL) and 200 μL PFP were then added as the inner aqueous phase (W1), which was sonicated for 6 min (work 5 s, and pause 5 s) using a sonicator (biosharp, FS-250N, China) to form an emulsion. Then, 5 mL 4% PVA-210 solution was added as the outer aqueous phase (W2), and continue to be sonicated for 6 min (work 5 s, and pause 5 s) to form a double emulsified solution. After that, 10 mL 2% isopropanol solution was added, and the solution was stirred at room temperature for 4 h to volatilize the dichloromethane. Finally, the PPUNPs solution was centrifugated at 13,000 rpm for 30 min, and washed with ultrapure water 3 times. The DiD label NPs were prepared in the similar method as PPUNPs. The only difference is that after 50 mg of PLGA was completely dissolved in 2 mL of dichloromethane 10 μL DiD (1 mg/mL in dichloromethane) was added into the PLGA solution as the organic phase (O).

### Preparation of thrombolytic biomimetic nano-drug carriers

#### Extraction of RBC membrane

Mouse whole blood was collected and placed in an ethylenediaminetetraacetic acid dipotassium salt dihydrate (EDTA.2 K) anticoagulant tube to prevent blood coagulation. Then 2 mL whole blood was centrifuged at 1000 rpm for 5 min, and serum was carefully removed. The blood was resuspended in 2 mL phosphate buffered saline (1×PBS) containing 1 mM EDTA.2 K. Repeated centrifugation and washing 3 times with PBS. Further, drop the RBCs solution into a hypotonic medium (0.25×PBS), and hypotonic at 4 °C for 30 min. Finally, the mixture was centrifuged at 10,000 rpm for 5 min to collect RBC membrane. A Bradford protein concentration assay kit was employed to analyze the total protein content in the obtained RBC membrane.

#### DSPE-PEG-RGD modified RBC membrane

The RBC membranes collected above were washed with ultrapure water by centrifugation, and then incubated with DSPE-PEG-RGD (20 μg/mL) for 30 min, and centrifuged at 8000 rpm for 5 min to obtain DSPE-PEG-RGD modified RBC membrane (RGD-RBC Membrane).

#### Preparation of RGD-RBCM@PPUNPs

RGD-RBCM@PPUNPs were fabricated by coating RGD-RBC Membrane on PPUNPs by a direct co-extrusion method. The RGD-RBC Membrane was sonicated in an ultrasonic bath (FS30D, 42 kHz, 100 W) for 5 min. Then, the sonicated RGD-RBC Membrane and PPUNPs were mixed at a ratio of membrane protein to polymer 1:1 (w/w). The mixture was extruded 10 times through a 400 nm polycarbonate porous membrane using an Avestin micro-extruder (Avestin, LF-1, Canada) to obtain RGD-RBCM@PPUNPs.

### Characterization of NPs

#### SEM and TEM observation

For SEM observation, appropriate nanoparticles were dissolved in double distilled water and then dropped on the conductive adhesive. After drying, the samples were sprayed with gold. The morphology, surface and dispersion were observed using field emission scanning electron microscopy (FESEM) at 5.0 kV. (JSM-7800F, Tokyo, Japan, JEOL). For TEM observation, a drop of NPs solution with a concentration of 100 μg/mL was deposited on a copper grid to be dried naturally and then stained with 1% phosphotungstic acid. High-resolution transmission electron microscopy (HRTEM) was used to observe the morphologies of PPUNPs and RGD-RBCM@PPUNPs by TEM under 200 kV (JEM-2100F, JEOL, Japan).

#### Measurement of particle size and Zeta potential

The particle size and Zeta potential of the NPs were measured by a Zetasizer Nano-ZS (Malvern, UK) at 25 °C using a nano-laser particle sizer (10 mV He-Ne laser light source, λ = 633 nm, incident angle 90°). All experimental operations were repeated at least 3 times.

### Determination of entrapment efficiency and drug loading rate of PPUNPs

#### Establishment of protein standard curve

The protein standard (Bovine serum albumin, BSA) with a concentration of 5 mg/mL was diluted different concentration gradients of 0, 0.125, 0.25, 0.5, 0.75, 1, and 1.5 mg/mL. 5 μL of protein standards of different concentrations were added to the 96-well plate. Then 250 μL of G250 staining solution was added to each well. The absorbance of each well was tested using a microplate reader at 595 nm, and then draw an absorbance-protein concentration standard curve was drawn according to the obtained data.

#### Determination of encapsulation efficiency and drug loading efficiency of UK

The content of UK in washing supernatant was measured by using a Bradford protein concentration assay kit and calculated according to the established protein standard curve. The quality of PPUNPs was measured after freeze-drying. Then, the encapsulation efficiency and drug loading efficiency of UK in PPUNPs were calculated as the following formula:

1.1 $$ER = \frac{{U_0 - U_1}}{{U_0}} \times 100\%$$

1.2 $$DR = \frac{{U_0 - U_1}}{{m_p}} \times 100\%$$

*ER*——Encapsulation rate, % *DR*——Drug loading rate, %*U*_*0*_——The amount of total urokinase, *U*_*1*_——Amount of free urokinase, *m*_*p*_——Quality of PPUNPs.

### In vitro ultrasound-triggered drug release of PPUNPs

PLGA nanoparticles loaded with UK (PUNPs) were used as the control group. PUNPs were prepared in the same method as PPUNPs. Take equal amounts of PUNPs and PPUNPs for freeze-drying. Then, the lyophilized powder of nanoparticles was added to 2 mL of PBS solution (pH = 7.4). The PUNPs and PPUNPs solution were placed in an ultrasonic cleaner to ultrasound treatment for 5 minutes. After ultrasound treatment, the NPs were performed to centrifugate. Then the released UK in the supernatant were measured by using the Bradford protein concentration assay kit (Biyuntian, China).

### Validation the modification of DSPE-PEG-RGD on RBC membrane and RGD

The collected RBC membranes were washed with ultrapure water. The red fluorescent probe DiI and DSPE-PEG-FITC were added to the RBC membrane solution and incubated for 30 min, then centrifuged at 8000 rpm for 5 min. The fluorescence was observed using a fluorescent microscope (Olympus, IX53, Japan).

### Protein characterization

The protein from different samples was extracted with native tissue/cell lysate kit (Solarbio, China). Then, the extracted protein samples were added to the loading wells of the SDS-PAGE gel, 20 μL per well. The gel was run at 75 V for 30 min, then the voltage was adjusted to 140 V until the protein band reached the bottom of the separating gel. Then, the gel was soak in Coomassie brilliant blue staining solution at room temperature for at least 4 h and the gel was decolorized by washing in Coomassie Brilliant Blue Destaining Solution until the bands were clear. Finally, the gel was imaged in a protein imaging system.

### In vitro hemolysis test

Fresh whole blood was collected from male New Zealand white rabbit via ear margin vein puncture using EDTA.2 K spray-coated tubes. 0.8 mL of anticoagulated rabbit blood mixed with 1 mL of PBS solution to prepare diluted anticoagulated rabbit blood. Then, 400 μL of different NPs in PBS at 1.5 mg/mL concentration was added to a 1.5 mL tube, PBS as the negative control group and ultrapure water as the positive control group. After incubation at 37 °C for 30 min, 100 μL of diluted anticoagulated rabbit blood was added to each sample, and incubated again at 37 °C for 60 min. Then the samples were centrifuged at 5000 rpm for 5 min, and the supernatants were collected. The absorbance of the supernatants from different samples was tested by a UV spectrophotometer (Shimadzu, UV-2600i, Japan) at 545 nm.

### In vitro cytotoxicity experiments

Endothelial cell lines and smooth muscle cell lines were purchased from the BeNa Culture Collection company. Endothelial cells and smooth muscle cells were grown in 1640 medium containing 10% fetal bovine serum (FBS) and 1% penicillin-streptomycin at 37 °C in a humidified atmosphere with 5% CO_2_. In in vitro cytotoxicity experiments, endothelial cells and smooth muscle cells were seeded at a density of 5 × 10^3^ cells per well in 96-well plates containing 200 μL medium of 10% FBS, 1% penicillin-streptomycin. After being cultured for 12 h, NPs were added to the culture plate at different concentrations. After incubation for 24 h, 10 μL of CCK-8 solution was added to each well, and the culture plate was put in the incubator for 2 h. Then, measure the absorbance at 450 nm was measured with a microplate reader (WAN XIANG, WX-SY96A, China), and the cell viability was calculated.

### In vitro macrophage phagocytosis

RAW 264.7 macrophages were purchased from the BeNa Culture Collection company. They were grown in DMEM medium supplemented with 10% FBS and 1% penicillin-streptomycin at 37 °C in a humidified atmosphere containing 5% CO_2_. In vitro uptake of NPs was assessed using RAW 264.7 macrophages. RAW 264.7 macrophages were seeded in a 24-well plate with cell slides at 2 × 10^5^ cells per well, and cultured in cell incubator at 5% CO_2_, 37 °C for 12 h. Then, 200 μL solution of different NPs was added. After incubation for 4 hours, the medium was removed and washed three times with PBS. Then the cells were fixed by 4% paraformaldehyde (Biyuntian, China) and stained by DAPI (Biyuntian, China). Finally, the uptake of NPs by RAW264.7 macrophages was observed using a laser scanning confocal microscope (Olympus, FV3000, Japan).

### In vitro thrombolysis

2 mL whole blood of the male New Zealand white rabbit was obtained via ear margin vein puncture and put into tube without anticoagulant. The fresh blood clots were achieved after blood clotted. Then, the blood clots were dried with filter paper, and weighed the initial mass. After weighing, the blood clots were put into tubes, and the solution of different NPs was added into each tube for 1 h incubation at 37 °C. The mass of the blood clots after reaction and drying with filter paper was weighed. The formula for calculating the thrombolysis rate is as follows:

1.3 $$TR = \frac{{W0 - W1}}{{W0}} \times 100\%$$

where: *TR*——Thrombolysis rate, % *W*_*0*_——Thrombosis initial mass, *W*_*1*_——Thrombosis mass after reaction.

## Results

### Preparation and characterization of PPUNPs

**Fig. 1:**
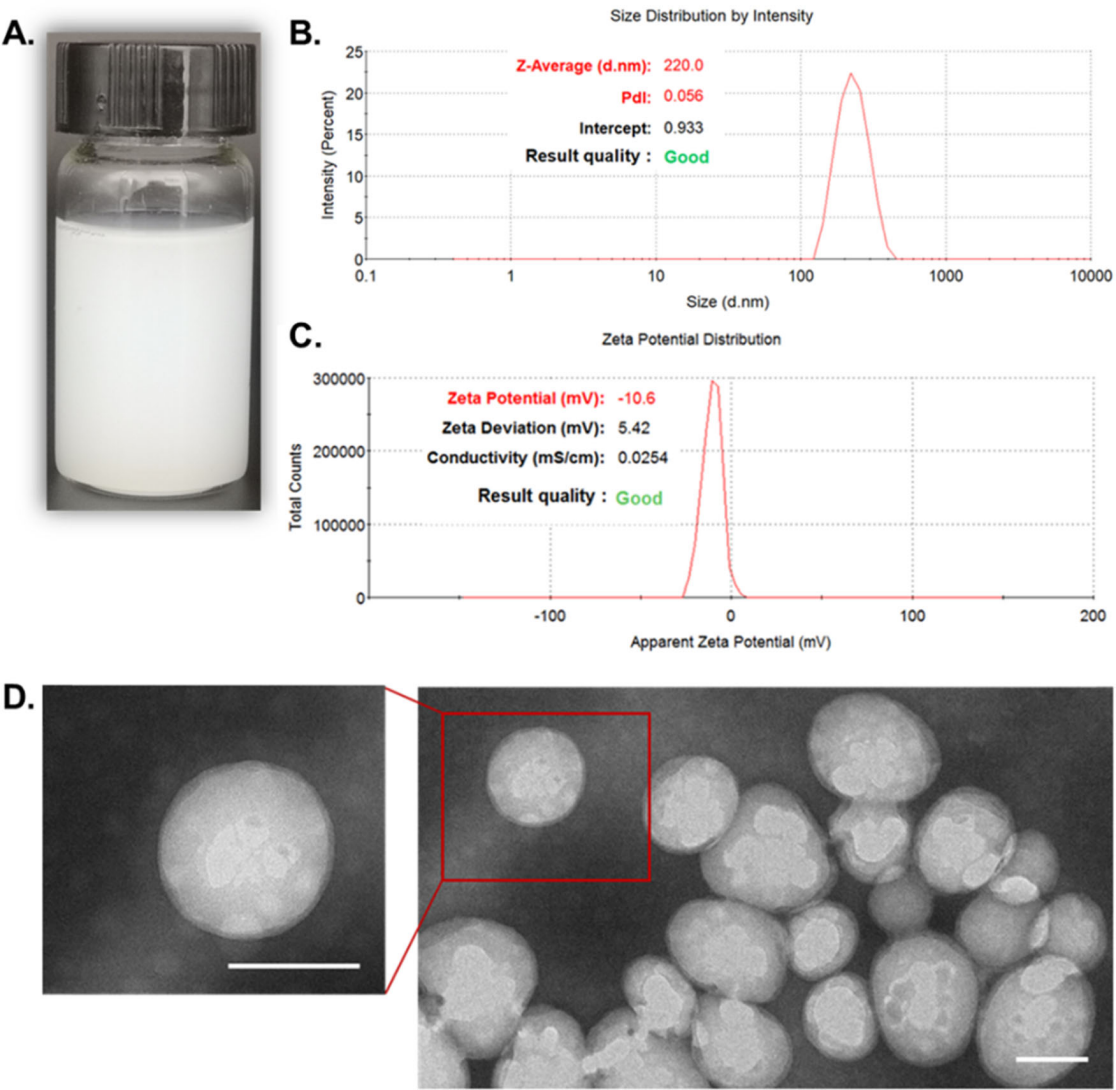
Characterization of PPUNPs. **A** The photo image of PPUNPs solution, **B** PPUNPs particle size distribution in aqueous solution, **C** Zeta potential of PPUNPs in aqueous solution, **D** TEM image of PPUNPs, Scale bar: 100 nm

The PPUNPs were prepared by the double-emulsion solvent evaporation method, and the PPUNPs solution was milky white, as shown in Fig. 1A. The particle size and surface Zeta potential of PPUNPs were determined by a Zetasizer nanoparticle size potentiometer. The average particle size of PPUNPs was 220.0 nm, and the poly dispersity index (PDI) was 0.056 (Fig. 1B). The surface Ztea potential of PPUNPs was −10.6 mV (Fig. 1C). The sample was further characterized by TEM, the morphology of PPUNPs was relatively uniform spherical (Fig. 1D). These results demonstrated that we successfully prepared the PPUNPs. In addition, PPUNPs have good dispersibility in aqueous solution, uniform distribution and negative surface potential.

### Encapsulation efficiency and drug loading efficiency of PPUNPs

Used Bradford protein concentration assay kit, the absorbance of BSA protein standard concentrations at 0, 0.125, 0.25, 0.5, 0.75, 1, 1.5 μg/mL were measured at 595 nm by microplate reader, and the absorbances were 0.569, 0.612, 0.665, 0.776, 0.863, 0.943, 1.101, respectively. The standard curve as shown in Fig. 1S. The standard curve equation was y = 0.3595x + 0.5783, R^2^ = 0.9952.

In order to determine the encapsulation efficiency and drug loading efficiency of UK in PUNPs and PPUNPs, the absorbance of the collected supernatants was measured at 595 nm with a microplate reader by using the Bradford protein concentration assay kit. The encapsulation efficiency was calculated according to formula (1.1), and the encapsulation efficiencies of UK in PUNPs and PPUNPs were 89.7% and 87.1%, respectively. The drug loading efficiencies of UK in PUNPs and PPUNPs were calculated according to formula (1.2), and were 5.92% and 6.14%, respectively (Table 1).

**Table 1:** Encapsulation efficiency and drug loading efficiency of UK in PUNPs and PPUNPs (*n* = 3)

Sample	Encapsulation efficiency	Drug loading efficiency
PUNPs	89.7%	5.92%
PPUNPs	87.1%	6.14%

### In vitro drug release of UK under ultrasound trigger

**Fig. 2:**
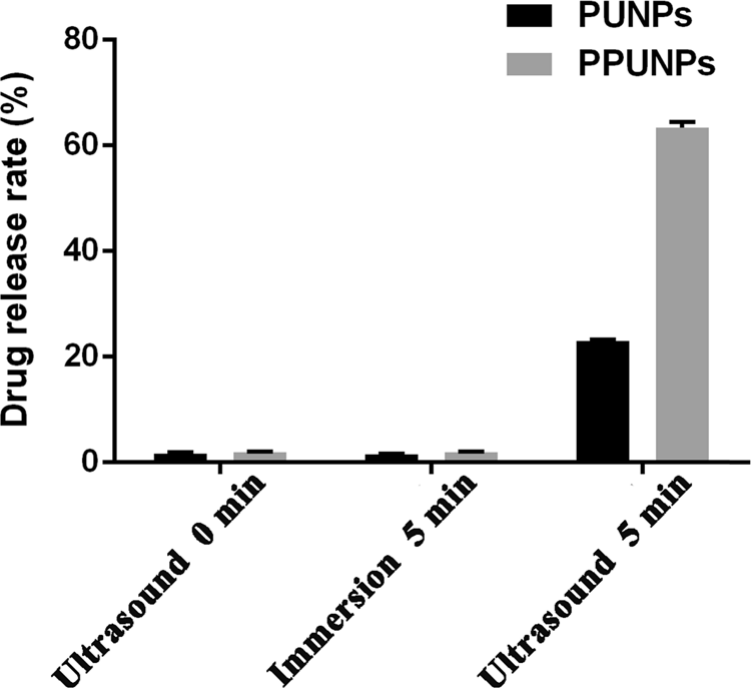
In vitro UK release rates of PUNPs and PPUNPs after 0, 5 min of ultrasound treatment or 5 min immersion in the solution without ultrasound treatment (*n* = 3)

PFP has the characteristics of liquid-gas phase transition. Therefore, PFP will change from liquid to gas state to facilitate drug release at elevated temperature or under the action of ultrasound [30]. As shown in Fig. 2, after 5 min of ultrasound trigger, the PPUNPs released about 60% UK, which was three times that of the PUNPs. Without ultrasound treatment or 5 min immersion in the solution without ultrasound treatment, both PUNPs and PPUNPs were almost no release of UK. This result showed that UK and PFP co-loaded into PLGA NPs could promote rapid release of UK from PPUNPs in response to ultrasound.

### DSPE-PEG-RGD modified RBC membrane

**Fig. 3:**
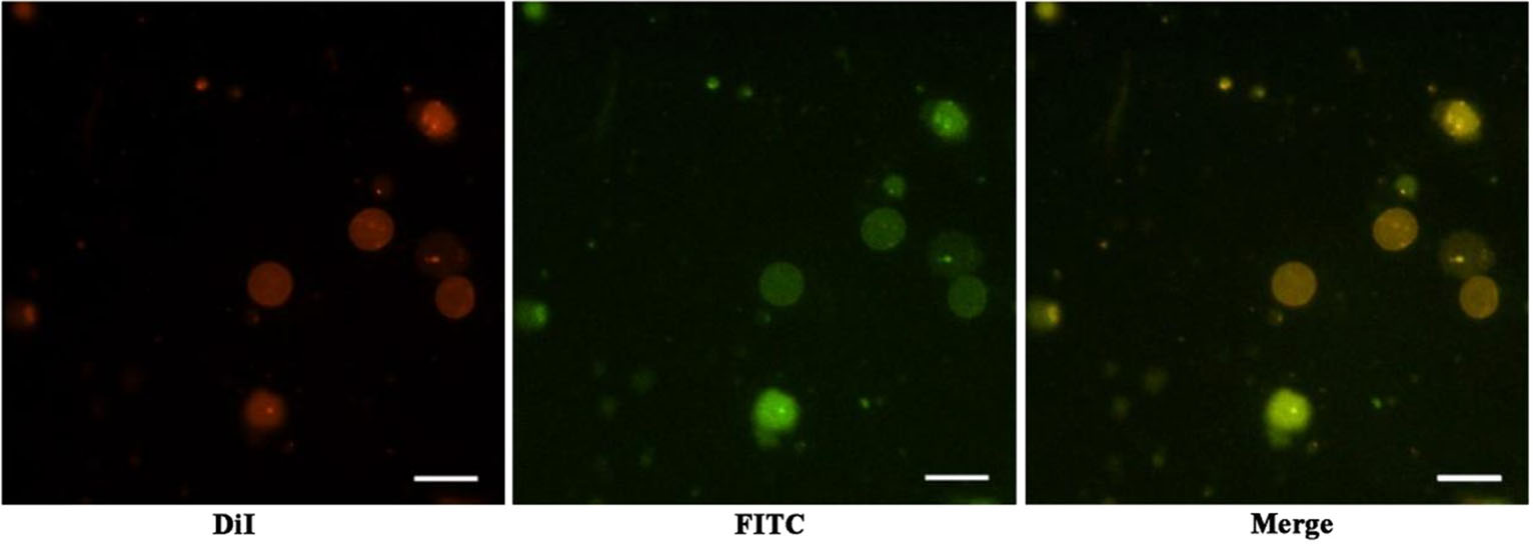
The fluorescence microscope image of DSPE-PEG-FITC modified and DiI labeled RBC membrane. (Scale bar: 10 μm)

Since DSPE-PEG-RGD don’t have fluorescent, DSPE-PEG-FITC was used to verify the feasibility of DSPE-PEG-RGD modification on RBC membrane by the insertion method. As shown in Fig. 3, DSPE-PEG-FITC (green) simultaneously appeared at the location of the DiI labeled RBC membrane (red). This result demonstrated that DSPE-PEG-RGD could be modified on the RBC membrane by the insertion method.

### Fabrication and characterization of RGD-RBCM@PPUNPs

**Fig. 4:**
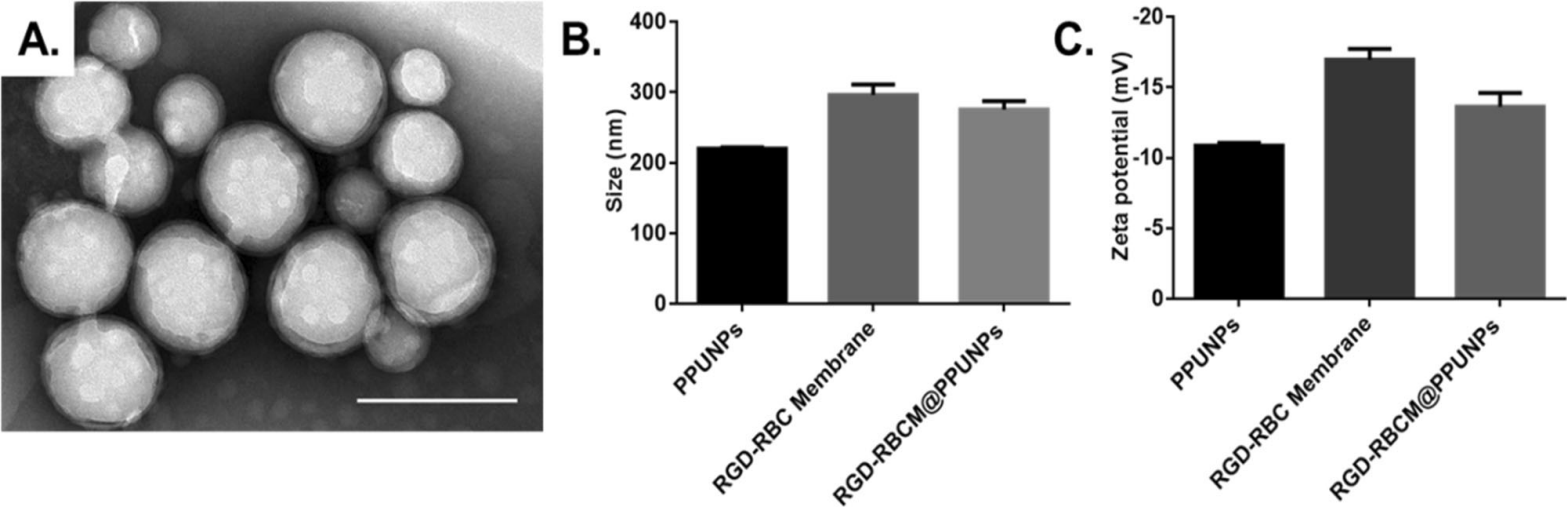
Characterization of RGD-RBCM@PPUNPs. **A** TEM image of RGD-RBCM@PPUNPs (scale bar=200 nm); **B** size and **C** Zeta potential of PPUNPs, RGD-RBC Membrane and RGD-RBCM@PPUNPs in aqueous solution (*n* = 3, mean ± SD)

RGD-RBC membrane was coated on the surface of PPUNPs nanoparticles by co-extrusion technology to prepare RGD-RBCM@PPUNPs. The RBC membrane coated on the surface of PPUNPs was detected by TEM. As shown in Fig. 4A, the RGD-RBCM@PPUNPs are spherical and have an obvious core-shell structure. Dynamic light scattering (DLS) analysis showed that the particle sizes of PPUNPs and RGD-RBC membrane in aqueous solution were 220.07 ± 2.12 nm (PDI: 0.051 ± 0.0312) and 296.33 ± 14.72 nm (PDI: 0.140 ± 0.0226) (Fig. 4B). After coated with the RGD-RBCM, the particle size of the RGD-RBCM@PPUNPs was 275.4 ± 12.13 nm (PDI: 0.147 ± 0.0436). Compared with the uncoated PPUNPs, the particle size of the RGD-RBCM@PPUNPs increased by 55.3 nm, which is due to the thickness of the RGD-RBC membrane coated on the surface of PPUNPs. The zeta potential of the RGD-RBC membrane was slightly reduced compared with the RBC membrane (Fig. S2). In addition, the surface zeta potentials of PPUNPs, RGD-RBC membrane, and RGD-RBCM@PPUNPs in aqueous solution were −10.87 ± 4.23 mV, −16.97 ± 5.26 mV and −13.63 ± 3.57 mV, respectively (Fig. 4C). The zeta potential of RGD-RBCM@PPUNPs was similar to RGD-RBCM. These results confirmed that the RGD-RBC membrane was coated on the surface of PPUNPs, and RGD-RBCM@PPUNPs were successfully prepared.

### Protein characterization

**Fig. 5:**
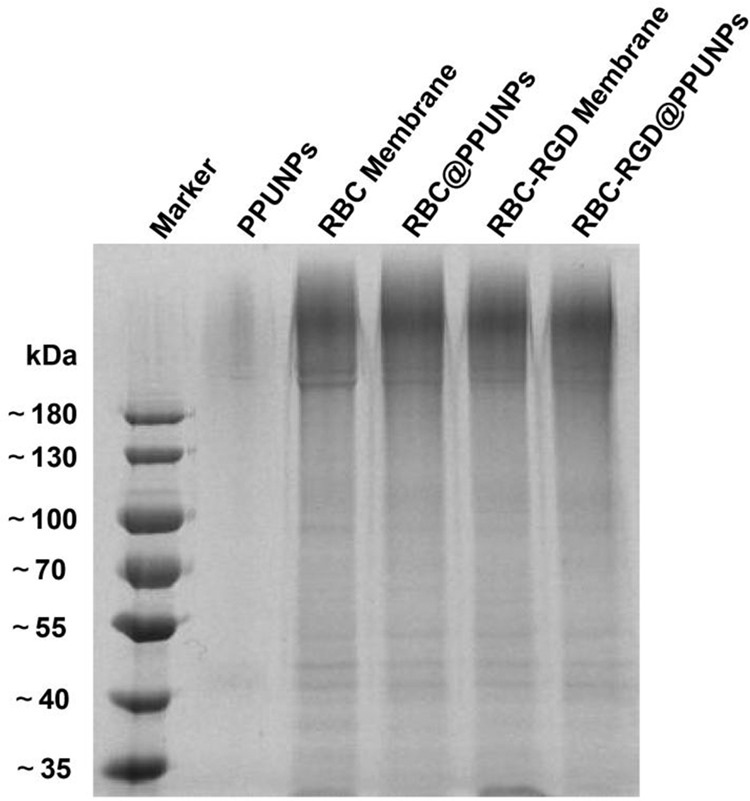
The SDS-PAGE gels images of PPUNPs, RBC membrane, RBCM@PPUNPs, RGD-RBC membrane and RGD-RBCM@PPUNPs

The biological functions of RGD-RBCM@PPUNPs mainly depend on various membrane proteins on the coated cell membrane. The proteins of PPUNPs, RBC membrane, RBCM@PPUNPs, RGD-RBC membrane and RGD-RBCM@PPUNPs were determined by SDS-protein gel experiments. As shown in Fig. 5, no protein bands appeared in PPUNPs, and the protein bands of RBC membrane, RBCM@PPUNPs, RGD-RBC membrane and RGD-RBCM@PPUNPs were coincident. The results indicated that the bilayer phospholipids of the RGD-modified red blood cell membrane and the corresponding membrane proteins can be successfully retained on the surface of the NPs.

### In vitro hemolytic effect analysis

**Fig. 6:**
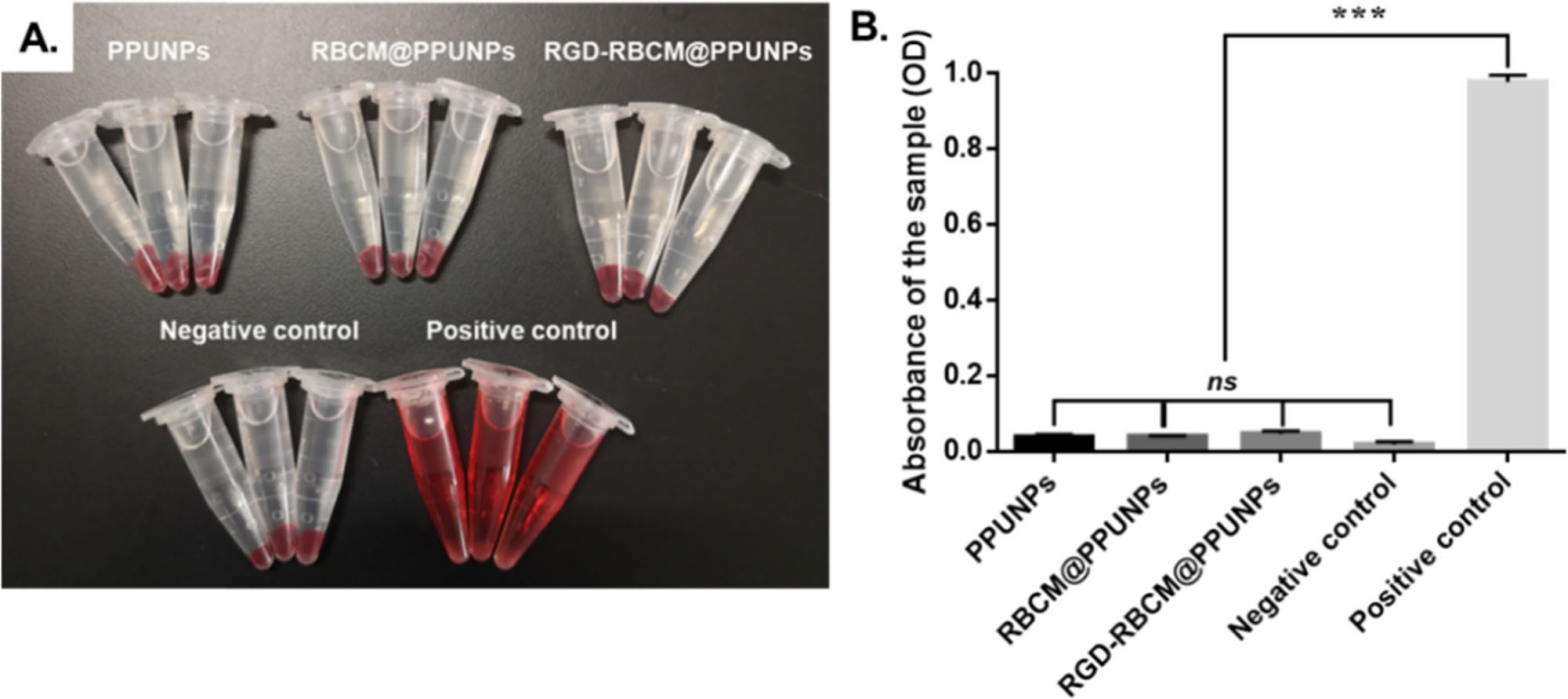
**A** The graph after centrifugation in the hemolysis experiment, **B** The absorbance after the hemolysis experiment (*n* = 3). ***p < 0.001, ns: no significant difference

The hemolytic effect of NPs was tested in vitro. As shown in Fig. 6A, after PPUNPs, RBCM@PPUNPs and RGD-RBCM@PPUNPs were incubated with fresh blood for a period of time, and the supernatant of the incubation solution after centrifugation was clear, indicating that the structure of RBCs was not destroyed. However, the solution of the positive control group was red after centrifugation, which indicates the RBCs were destroyed and occurred hemolysis. Moreover, the OD value of each group was measured at 545 nm by UV spectrophotometer. There was no significant difference between the RGD-RBCM@PPUNPs and the negative control group (Fig. 6B). The results demonstrated that RGD-RBCM@PPUNPs did not cause the destruction of RBCs and had good blood compatibility.

### In vitro cytotoxicity effects

**Fig. 7:**
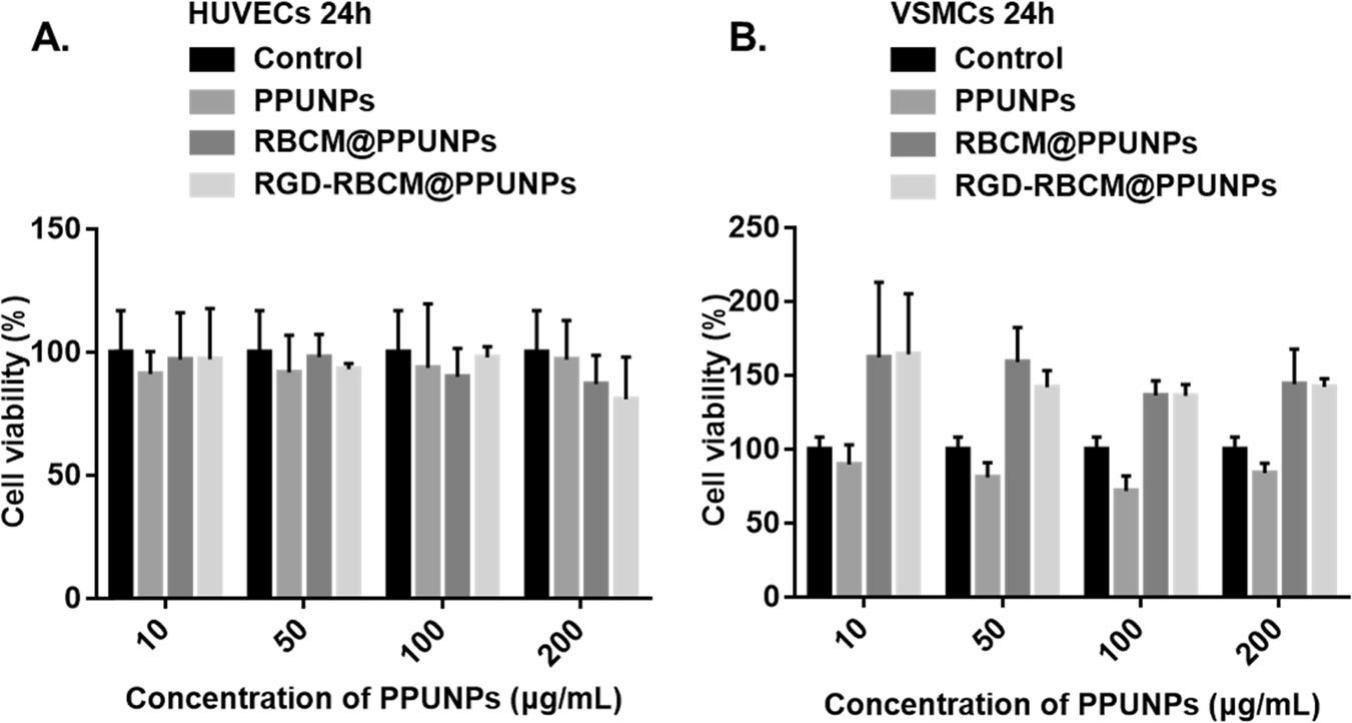
The cell viability of endothelial cells (**A**) and smooth muscle cells (**B**) after treatment with PPUNPs, RBCM@PPUNPs, or RGD-RBCM@PPUNPsfor 24 h (*n* = 3)

The proliferation ability of endothelial cells and smooth muscle cells under PPUNPs, RBCM@PPUNPs and RGD-RBCM@PPUNPs treatment were evaluated by in vitro cytotoxicity experiments. As shown in Fig. 7A, B, compared with the control group, PPUNPs, RBCM@PPUNPs and RGD-RBCM@PPUNPs had no obvious cytotoxicity to endothelial cells and smooth muscle cells within 24 h treatment, which indicates that PPUNPs, RBCM@PPUNPs and RGD-RBCM@PPUNPs had good cytocompatibility.

### Macrophage uptake

**Fig. 8:**
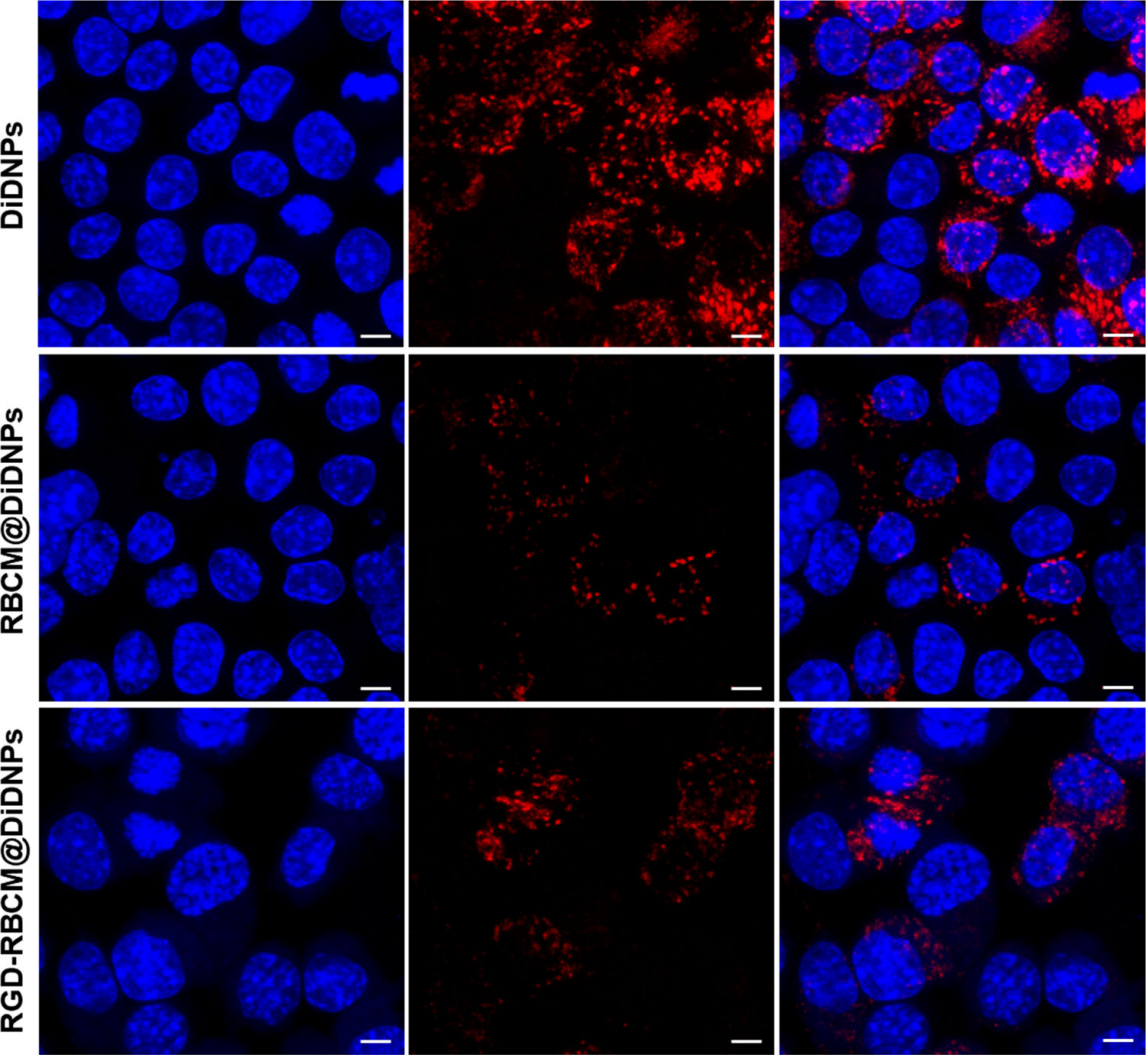
Confocal laser scanning microscope images of macrophages incubated with DiDNPs, RBCM@DiDNPs and RGD-RBCM@DiDNPs for 4 h. DAPI-stained nuclei (blue), NPs (red). (Scale bar: 5 μm.)

Previous studies demonstrated that the coating of RBC membranes could inhibit the uptake of NPs by immune cells [18, 31]. Therefore, the uptake of RGD-RBCM@PPUNPs was examined in vitro using RAW 264.7 cells. In order to facilitate fluorescence observation, the NPs were labeled with DiD. As shown in Fig. 8, after macrophages were incubated with DiDNPs, RBCM@DiDNPs or RGD-RBCM@DiDNPs for 4 h, stronger red fluorescence from the DiDNPs group was detected than the fluorescence from RBCM@DiDNPs and RGD-RBCM@DiDNPs, which indicated that DiDNPs were significantly internalized by macrophages, while RBCM@DiDNPs and RGD-RBCM@DiDNPs RGD-RBCM@DiDNPs could inhibit the phagocytosis of macrophages. Those results demonstrate that RBC membranes coated NPs can effectively inhibit internalization by macrophages, which is a great benefit to prolong their blood circulation time.

### In vitro thrombolysis

**Fig. 9:**
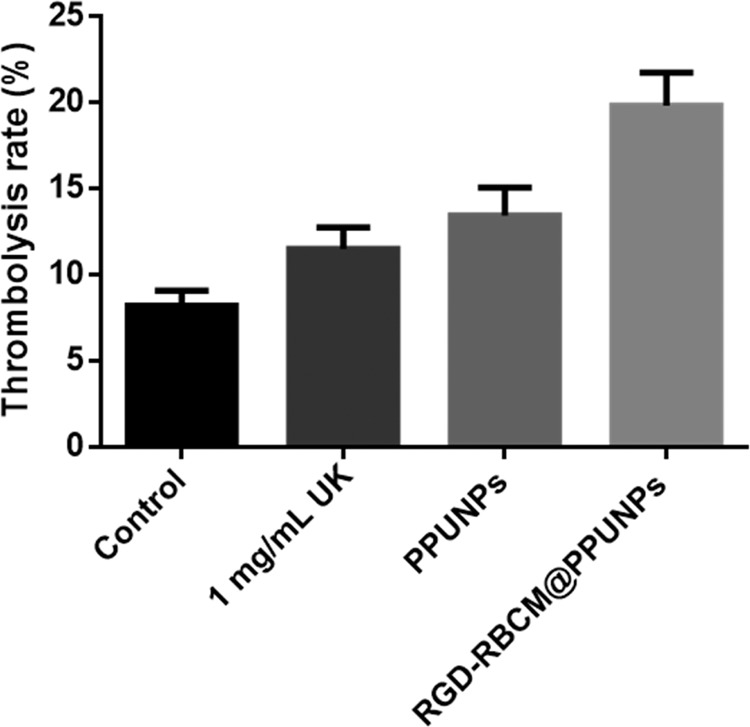
Statistical chart of in vitro thrombolysis rates of PBS, UK, PPUNPs and RGD-RBCM@PPUNPs (*n* = 3)

Subsequently, the thrombolysis effect of RGD-RBCM@PPUNPs was evaluated in vitro. As shown in Fig. 9, blood clots were incubated with UK, PPUNPs, or RGD-RBCM@PPUNPs at the concentration of 1 mg/mL UK. After 1 h treatment, the thrombolysis rate was calculated according to formula (1.3). The thrombolysis rates of UK, PPUNPs and RD-RBCM@PPUNPs were 11.5%, 13.5% and 19.8%, respectively (Table 2). Compared with UK and PPUNPs, the thrombolysis rate of RGD-RBCM@PPUNPs increased by 8.3% and 6.3%, respectively. This result showed that RGD-RBCM@PPUNPs had a more efficient thrombolysis effect.

**Table 2:** In vitro thrombolysis rate (*n* = 3)

Samples	Control	1 mg/mL UK	PPUNPs	RGD-RBCM@PPUNPs
Thrombolysis rate	8.2%	11.5%	13.5%	19.8%

## Discussion

At present, thrombolytic targeting strategies mainly include the use of ultrasound or magnetic field as external stimulation conditions to guide the drug carrier to the thrombus site to play a role, or to stimulate the specific binding of drug carrier and thrombus components to achieve the purpose of treatment [32, 33]. For example, Zhou et al. constructed Fe3O4-based PLGA nanoparticles (NPs) carrying recombinant tissue plasminogen activator (rtPA) (Fe3O4–PLGA-rtPA/CS-cRGD), which further coated with cyclic arginine-glycine-aspartic peptide (cRGD) grafted chitosan (CS) [34]. The fabricated Fe3O4–PLGA-rtPA/CS-cRGD NPs can be used as a dual-function tool in the early detection of a thrombus and in the dynamic monitoring of the thrombolytic efficiency using MRI. Zhong et al. encapsulated PFH droplets in PLGA NPs and further loaded Fe3O4 NPs on the surface [35]. Co-assembly fibrin-targeting peptide-decorated nanoassembly DiR and ticagrelor (FT-DT NPs) for thrombus-homing delivery, Ft-dt NPs can display fluorescence signal at the site of thrombi, meanwhile have photothermal/antiplatelet synergistic thrombolytic effect in vivo [36]. In this study, we fabricated a biomimetic nano-thrombolytic drug RGD-RBCM@PPUNPs for targeted thrombus treatment. Firstly, the PPUNPs were successfully prepared by the double-emulsion solvent evaporation (Fig. 1), which have nanoscale with 220 nm, negative surface potential, good dispersibility, and uniform distribution. Then, we carried out in vitro drug release experiments to investigate the release of UK under ultrasound treatment. As shown in Fig. 2, ultrasound treatment for 5 min can greatly increase the release of UK from PPUNPs, which contrasts markedly with the control group. Moreover, without ultrasound treatment, both PUNPs and PPUNPs were almost no release of UK.

For nanomedicine, good biocompatibility and low biotoxicity are essential basic properties [37, 38]. The use of autologous cell membranes to modify nanomaterials has been a common approach in recent years [31, 39]. Red blood cell membrane, platelet membrane or macrophage membrane were generally selected, both of which were easy to extract and have good biocompatibility [40]. Studies have shown that the affinity of nano-drugs is enhanced after being wrapped by cell membrane, which can not only prolong blood circulation, also enhance tumor tissue penetration in cancer treatment [41]. The functional modification of platelet membrane on nanoparticles can achieve multifactorial biological targeting and detection of atherosclerosis [42]. Fe3O4@M coated with macrophage membrane is regarded as a promising contrast agent for the diagnosis of early atherosclerosis [43]. Platelet-coated PM@Se/Rb1 NPs, in addition to improving inflammatory behavior, such as effectively reducing adhesion and inhibiting angiogenesis, is also effective in the localization of atherosclerotic plaques [44]. In this study, we used RBC membrane to coat on the surface of PPUNPs. In addition, in order to improve the targeted ability of NPs to thrombus, the RBC membranes were further modified with RGD peptide through the insert method. As shown in Fig. 3, after treating with DSPE-PEG-FITC, the green fluorescence (DSPE-PEG-FITC) coincided with the red fluorescence (DiI labeled RBC membrane), which indicated that DSPE-PEG-RGD could be modified on the RBC membrane by the insertion method. The RGD modified membranes were coated on PPUNPs with the co-extrusion method. The fabricated RGD-RBCM@PPUNPs were demonstrated by TEM, particle sizes detection, zeta potential detection and protein analysis (Figs. 4 and 5), which confirmed that the RGD-RBC membrane was successfully coated on the surface of PPUNPs. Moreover, in vitro experiments showed that RGD-RBCM@PPUNPs possessed good blood compatibility (Fig. 6) and cell compatibility (Fig. 7). After the structure and compatibility had been verified, we evaluated the potential of RGD-RBCM@PPUNPs for thrombosis treatment. We investigated the level of internalization by macrophages to demonstrate that RGD-RBCM@PPUNPs have the potential to escape from clearance by the immune system (Fig. 8). Importantly, in vitro thrombolytic experiments indicated that RGD-RBCM@PPUNPs showed a higher thrombolytic rate than other experimental groups (Fig. 9).

We conducted a series of tests on RGD-RBCM@PPUNP, which effectively proved its good biocompatibility, blood circulation ability and targeted therapeutic effect in vitro. However, there are still some differences between in vivo and in vitro, which cannot be compensated for in vitro experiments. In future work, the efficacy of RGD-RBCM@PPUNP for targeted thrombolysis therapy should be investigated in vivo, which is conducive to promote RGD-RBCM@PPUNP became a potential strategy for targeted thrombolysis therapy.

## Conclusion

In this study, we constructed a biomimetic targeted thrombolysis nanomedicine RGD-RBCM@PPUNPs by cell membrane coating technology, which shown good nanomedicine properties, hemocompatibility and cytocompatibility. In in vitro experiments, it can inhibit the phagocytosis of macrophages. In addition, RGD-RBCM@PPUNPs has more efficient thrombolysis effect than free UK and uncoated NPs. In general, this study provides a potential strategy for targeted thrombolysis therapy.

## Supplementary Information

Supporting Information
