# Supplementary material for: RGD peptide modified RBC membrane functionalized biomimetic nanoparticles for thrombolytic therapy
Source: J Mater Sci Mater Med. 2023 Apr 12;34(4):18. doi: 10.1007/s10856-023-06719-1 (PMC10097782; doi:10.1007/s10856-023-06719-1)
Supplement: Supplementary file 1 — Supporting Information [file 10856_2023_6719_MOESM1_ESM.docx]

**Supporting Information**

**RGD** **peptide modified RBC membrane functionalized** **biomimetic nanoparticles for thrombolytic therapy**

Zichen Xu^1^, Jinxia Huang^3^ Tao Zhang^3^, Wenfeng Xu^3^, Xiaoling Liao^3^, Yi Wang^2*^, Guixue Wang^1*^

^1^Key Laboratory for Biorheological Science and Technology of Ministry of Education, State and Local Joint Engineering Laboratory for Vascular Implants, Bioengineering College of Chongqing University, Chongqing, 400030, China

^2^College of Basic Medical Sciences, Chongqing Medical University, Chongqing 400016, China

^3^Chongqing Key Laboratory of Nano/Micro Composite Material and Device, School of Metallurgy and Materials Engineering, Chongqing University of Science and Technology, Chongqing, 401331, China

*Corresponding authors:

Guixue Wang, Key Laboratory for Biorheological Science and Technology of Ministry of Education, State and Local Joint Engineering Laboratory for Vascular Implants, Bioengineering College of Chongqing University, Chongqing, 400030, China. Email：[wanggx@cqu.edu.cn](mailto:wanggx@cqu.edu.cn)

Yi Wang, College of Basic Medical Sciences, Chongqing Medical University, Chongqing 400016, China. Email: [wangyi@cqmu.edu.cn](mailto:wangyi@cqmu.edu.cn)


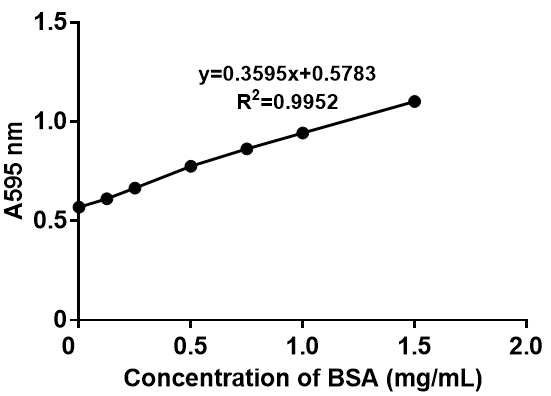


**Fig. S1** The standard curve of BSA protein at different concentrations





**Fig. S2** The zeta potential of the RBC membrane and RGD modification RBC membrane
